# The Influence of Treatment with PCSK9 Inhibitors and Variants in the *CRP* (rs1800947), *TNFA* (rs1800629), and *IL6* (rs1800795) Genes on the Corresponding Inflammatory Markers in Patients with Very High Lipoprotein(a) Levels

**DOI:** 10.3390/jcdd9050127

**Published:** 2022-04-22

**Authors:** Tina Levstek, Nik Podkrajšek, Andreja Rehberger Likozar, Miran Šebeštjen, Katarina Trebušak Podkrajšek

**Affiliations:** 1 Institute of Biochemistry and Molecular Genetics, Faculty of Medicine, University of Ljubljana, Vrazov trg 2, 1000 Ljubljana, Slovenia; tina.levstek@mf.uni-lj.si (T.L.); nik.podkrajsek@dijaki.gimb.org (N.P.); 2 Department of Vascular Diseases, University Medical Centre Ljubljana, Zaloška cesta 7, 1000 Ljubljana, Slovenia; andreja.rehbergerlikozar@kclj.si (A.R.L.); miran.sebestjen@guest.arnes.si (M.Š.); 3 Department of Cardiology, University Medical Centre Ljubljana, Zaloška cesta 7, 1000 Ljubljana, Slovenia; 4 Department of Internal Medicine, Faculty of Medicine, University of Ljubljana, Zaloška cesta 7, 1000 Ljubljana, Slovenia; 5 Clinical Institute for Special Laboratory Diagnostics, University Children’s Hospital, University Medical Centre Ljubljana, Vrazov trg 1, 1000 Ljubljana, Slovenia

**Keywords:** polymorphism, inflammation, *CRP* rs1800947, *TNFA* rs1800629, *IL6* rs1800795, lipoprotein(a), PCSK9 inhibitors, cardiovascular disease

## Abstract

Chronic inflammation contributes significantly to the development and progression of atherosclerosis. However, the factors that lead to an inflammatory imbalance towards a proinflammatory state are not yet fully understood. The *CRP* rs1800947, *TNFA* rs1800629, and *IL6* rs1800795 polymorphisms may play a role in the pathogenesis of atherosclerosis and were therefore selected to investigate the influence of genetic variability on the corresponding plasma levels after treatment with a proprotein convertase subtilisin/kexin type 9 (PCSK9) inhibitor. A group of 69 patients with stable coronary artery disease after myocardial infarction before the age of 50 years and very high lipoprotein(a) levels were enrolled in the study. All patients received a PCSK9 inhibitor (evolocumab or alirocumab). Genotyping was performed using TaqMan assays (*CRP* rs1800947, *TNFA* rs1800629, and *IL6* rs1800795). Consistent with previous studies, no significant change in levels of inflammatory biomarkers was observed after 6 months of treatment with PCSK9 inhibitors. We also did not detect any significant association between single nucleotide polymorphisms *CRP* rs1800947, *TNFA* rs1800629, and *IL6* rs1800795 and plasma levels of high-sensitivity C-reactive protein (hsCRP), tumor necrosis factor-α (TNF-α), or interleukin 6 (IL6), respectively, at enrollment. However, the difference in IL6 levels after treatment with PCSK9 inhibitors was statistically significant (*p* = 0.050) in patients with *IL6*-74CC genotype, indicating the possible role of the *IL6* rs1800795 polymorphism in modulating inflammation.

## 1. Introduction

Atherosclerosis was considered to be the degenerative disorder of the vessel wall, with elevated levels of low-density lipoprotein cholesterol (LDL-C) being one of the best-known risk factors for cardiovascular disease (CVD). However, there is growing evidence that inflammation plays a central role in all steps of atherogenesis, from endothelial dysfunction, plaque formation, its rupture and thrombus formation [1]. Proinflammatory cytokines such as interleukin (IL) 6 and tumor necrosis factor-α (TNF-α) promote inflammation, whereas anti-inflammatory cytokines such as IL10 attenuate the inflammatory response. An impaired inflammatory balance with a shift toward a proinflammatory state leads to chronic low-grade inflammation, in which gene polymorphisms may play an important role [2].

High-sensitivity C-reactive protein (hsCRP) is a well-established marker of inflammation and is associated with risk for CVD [3]. However, its casual role in the development of CVD is questionable [4]. CRP is produced in the liver in response to proinflammatory cytokines such as IL6, which is secreted by activated cells at the site of inflammation [5]. CRP levels are considered to be an independent predictor of acute cardiovascular events in primary [6] and secondary prevention [7]. CRP levels are determined by several factors such as age, body mass index, and smoking status [8]. In addition, there is also evidence of a strong genetic component, as family and twin studies have shown that genetic variability in the *CRP* gene accounts for up to 40% of the variance in CRP levels [9,10,11]. The most studied variant of the *CRP* gene is rs1800947, a synonymous substitution within exon 2 known as G1059C, which results in lower CRP levels [12].

Single nucleotide polymorphisms (SNPs) of cytokine genes affect gene transcription and cytokine secretion that could modulate the risk of CVD [12]. TNF-α is one of the most important proinflammatory cytokines. Through its presence in atherosclerotic plaques, TNF-α contributes to plaque progression by enhancing the local inflammatory response [13,14]. Substitution in the promoter region of the *TNFA* gene (rs1800629), known as −308G>A, leads to increased TNF-α production [15]. The proinflammatory cytokine IL6 modulates endothelial synthase activity in endothelial cells, involved in leukocyte recruitment to the vessel wall, and stimulates smooth muscle cell proliferation [16]. The rs1800795 polymorphism in the *IL6* gene, known as −174G>C, is a substitution in the promoter region affecting the transcription rate of the gene and subsequent plasma concentrations of IL6 [17]. However, the effect of this polymorphism is complex and may depend on other factors such as physiological and psychological stress, metabolic factors, age, and body mass index [18]. In patients after acute myocardial infarction IL6 levels have been associated with increased cardiovascular mortality during a 3-year follow-up period independently of other inflammatory parameters [19].

In addition to inflammatory markers, elevated lipoprotein(a) (Lp(a)) levels have been identified as one of the major contributors to atherosclerotic changes and as an independent predictor of cardiovascular morbidity and mortality [20] in the general population and in patients with established coronary artery disease [21]. The proinflammatory effects of Lp(a) are mediated by increased oxidized phospholipid content in Lp(a), induced expression of inflammatory cytokines and ILs, including IL1β, IL6, IL8, and TNF-α, and increased chemotaxis of monocytes [22].

The level of Lp(a) is genetically determined by variants in the *LPA* gene with approximately 30% of the population having levels in the atherothrombotic range [23]. The incidence among patients with established CVD is possibly even higher [24]. Pharmacological interventions to lower LDL-C levels are well established, whereas effective Lp(a)-lowering therapies are still in development [25]. Proprotein convertase subtilisin/kexin type 9 (PCSK9) inhibitors reduce LDL-C levels by up to 65%, while the reduction in Lp(a) ranges from 20 to 40%; therefore, in patients with very high Lp(a) levels, Lp(a) remains elevated [26].

Inflammation is not only the driver of CVD, but also represents a possible therapeutic target. Complex mechanisms underlying the atherosclerotic inflammation and variability between different subjects should be elucidated. Since the genetic component may have an important role, the objectives of our study were (1) to determine the allelic frequencies and genotype distribution of polymorphisms *CRP* rs1800947, *TNFA* rs1800629, and *IL6* rs1800795; (2) to identify the association of these polymorphisms on inflammatory status; and (3) to evaluate the effect of genetic variability and 6 months of treatment with PSCK9 inhibitors on inflammatory status in a cohort of patients with myocardial infarction before the age of 50 years and very high Lp(a) levels.

## 2. Materials and Methods

### 2.1. Study Participants

A total of 69 patients were enrolled in the study from November 2019 to May 2021 and were followed for 6 months. All patients had clinically stable coronary artery disease for at least 6 months after myocardial infarction, which occurred before the age of 50 years. Included patients had a Lp(a) level > 1000 mg/L or Lp(a) level > 600 mg/L and LDL-C level > 2.6 mmol/L. All patients received maximally tolerated statin therapy and ezetimibe if needed, β-blockers, antiplatelet drugs, and angiotensin-converting enzyme inhibitors. Their therapy had not changed for at least 2 months before enrollment in the study. The main exclusion criteria were liver transaminases elevated more than three times above reference values, severe renal dysfunction, serum creatinine level higher than 200 mmol/L, and acute illness in the previous 6 weeks.

After enrolment, all patients underwent clinical and laboratory evaluation and were then randomized into two groups. The first group of 39 patients received a PCSK9 inhibitor (alirocumab (150 mg) or evolocumab (140 mg), subcutaneously, every two weeks), whereas the second group of 30 patients received placebo initially, and after six months, the PCSK9 inhibitor. In total, 35 patients received alirocumab and 34 received evolocumab. Clinical and laboratory parameters were measured before and after six months of placebo and after six months of the treatment period as previously described in detail [27].

The study protocol was approved by the Slovenian Ethics Committee for Research in Medicine (0120-357/2018/8). All participants gave written informed consent in accordance with the Declaration of Helsinki.

### 2.2. Blood Collection and Laboratory Investigations

Peripheral blood samples were collected in tubes without and with anticoagulant (K3-EDTA) to obtain serum, plasma, and buffy-coat. Aliquots of samples were immediately stored at –20 °C. All laboratory parameters were measured in the Laboratory of Haemostasis and Atherothrombosis at the Clinical Department of Angiology, University Clinical Centre Ljubljana, according to standard laboratory procedures.

### 2.3. Genotyping

Genomic DNA was isolated from buffy-coat samples using the FlexiGene DNA kit (Qiagen, Hilden, Germany) according to the manufacturer’s instructions. Three SNPs were selected for analysis: *CRP* rs1800947, *TNFA* rs1800629, and *IL6* rs1800795. Genotyping was performed using Predesigned TaqMan SNP Genotyping Assays (Thermo Fisher Scientific, Waltham, MA, USA) and TaqMan™ Genotyping Master Mix (Applied Biosystems, Waltham, MA, USA) according to the manufacturer’s instructions in a QuantStudio 7 Flex Real-Time PCR System (Applied Biosystems, Waltham, MA, USA). Genotyping was performed blind to all clinical data and was repeated randomly on 15% of samples to check the reliability of genotyping.

### 2.4. Statistical Analysis

All analyses were performed using IBM SPSS Statistics version 27.0 (IBM Corporation, New York, NY, USA). For descriptive statistical analysis, the normality of the distribution of continuous variables was tested using Shapiro–Wilk test. The median with interquartile range (25–75%) or the mean with standard deviation were used to describe the central tendency and variability of the continuous variables. The frequencies were used to describe the distribution of the categorical variables. Wilcoxon signed-rank test was used to assess changes during the placebo and treatment periods. For all SNPs, Chi-square test was used to assess deviation from Hardy–Weinberg equilibrium (HWE). The association of genotyping data with continuous variables were analyzed using the nonparametric Mann–Whitney U tests. All statistical tests were two-sided. The level of statistical significance was set at 0.05. Figures were prepared with GraphPad Prism 9 (San Diego, CA, USA).

## 3. Results

### 3.1. Patient Characteristics

A total of 69 patients with a median age of 52.6 (45.8–56.9) years and a mean age of 45 years at first coronary event were included in the study. The cohort was composed of 61 (88.4%) men and 8 (11.6%) women. Patient characteristics and levels of lipid parameters and inflammatory markers are shown in Table 1.

### 3.2. Single Nucleotide Polymorphism Frequencies in Selected Genes of Inflammatory Markers

The distribution of genotype frequencies for the polymorphisms studied and minor allele frequencies (MAFs) are shown in Table 2. All SNPs were in HWE (*p* > 0.05). MAFs for the European non-Finnish population, retrieved from the gnomAD database v2.1.1 (https://gnomad.broadinstitute.org/, accessed on 20 January 2022), are 0.065 for *CRP* rs1800947, 0.443 for *IL6* rs1800795, and 0.165 for *TNFA* rs1800629 and are in agreement with our cohort.

### 3.3. Plasma Levels of Inflammatory Markers at Enrollment and after Treatment with PCSK9 Inhibitors

Plasma levels of inflammatory markers were measured at enrollment and after 6 months of placebo or treatment. Plasma levels of hsCRP, TNF-α, IL6, and IL8 did not change significantly in either the control or treatment groups (Table 3).

### 3.4. The Influence of Investigated Genotypes on Plasma Levels of Inflammatory Markers and Levels of Lipoprotein(a)

The major novelty of our study was that we examined the association between genotypes and plasma levels of inflammatory markers. For all polymorphisms, we used an additive genetic model, in which we combined patients with a present alternate allele. There was no statistically significant difference between the different genotypes and the levels of the corresponding inflammatory markers (Table 4).

Additionally, polymorphisms *TNFA* rs1800629 and *IL6* rs1800795 were not associated with hsCRP levels (*p* = 0.467 and *p* = 0.741, respectively).

**Figure 1:**
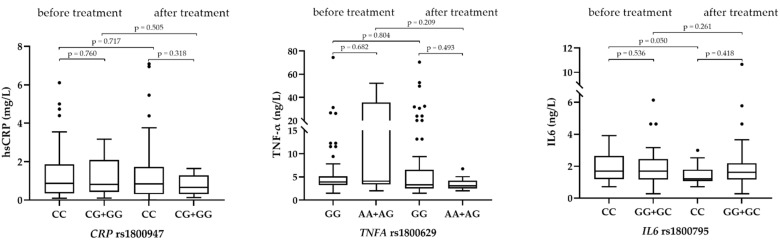
Association of genotypes with plasma levels of inflammatory markers before and after treatment (*N* = 68). Black dots represents outliers.

There was no statistically significant difference in hsCRP and TNF-α levels before and after 6 months of treatment, regardless of the genotype present. However, IL6 levels were significantly different (*p* = 0.050) in patients with genotype CC after treatment with PCSK9 inhibitors (Figure 1).

The variability in inflammatory genes was not associated with the level of Lp(a) at enrolment in the study (Table 5).

### 3.5. The Correlation of the Change of Inflammatory Markers with the Change of Lipid Parameters after Treatment with PCSK9 Inhibitors

We performed Spearman’s Rho correlation analysis to investigate the association between the change in plasma level of inflammatory markers and lipid parameters after 6 months of treatment with PCSK9 inhibitors. We found no statistically significant correlations between the change in hsCRP, TNF-α, IL6, and IL8 levels and the change in lipid parameters (Table 6).

**Figure 2:**
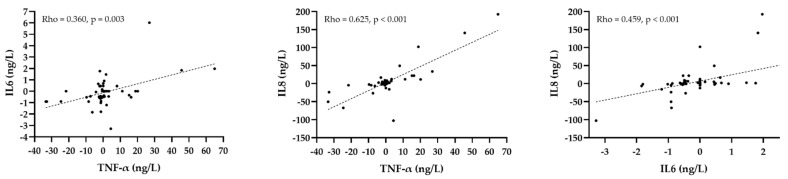
Correlation between changes in plasma cytokine levels before and after treatment with PCSK9 inhibitors. Black dots represent each patient, whereas the dashed line indicates the regression line.

However, we found significant correlations between changes in some plasma cytokine levels. The change in plasma TNF-α level was correlated with the change in IL6 (Rho = 0.360, *p* = 0.003) and IL8 (Rho = 0.625, *p* < 0.001). There was also a correlation between the change in IL6 and IL8 (Rho = 0.459, *p* < 0.001) (Figure 2).

## 4. Discussion

To our knowledge, the present study is the first to investigate *CRP* rs1800947, *TNFA* rs1800629, and *IL6* rs1800795 polymorphisms and their association with the corresponding plasma levels after treatment with PCSK9 inhibitors. Our results showed no significant difference in hsCRP and TNF-α levels before and after treatment, regardless of the genotype present. On the other hand, *IL6* rs1800795 CC polymorphism was associated with a statistically significant difference in IL6 levels after treatment with PCSK9 inhibitors. This has not been reported previously and is suggesting that the G allele of the *IL6* rs1800795 polymorphism is likely to be associated with increased residual inflammation after treatment with PCSK9 inhibitors in patients with premature myocardial infarction and very high Lp(a) levels. Chronological measurements allowed us not only to assess the influence of genetic variability at the baseline levels, but also to evaluate the impact of genetic variability on the plasma levels of inflammatory markers after treatment with PCSK9 inhibitors. Indeed, the study of gene polymorphisms in inflammatory marker genes could help to elucidate the dynamics of the inflammatory response and to develop new therapeutic strategies. However, the studies of gene polymorphisms in relation to inflammatory markers after treatment with PCSK9 inhibitors are scarce. Several clinical trials in CVD patients have investigated the effect of PCSK9 inhibitors on inflammatory markers, especially on hsCRP levels. A multicenter, double-blind, placebo-controlled study EQUATOR showed that 24 weeks of treatment with the PCSK9 inhibitor RG7652 did not significantly alter circulating hsCRP levels and the proinflammatory cytokines TNF-α and IL6 in patients at high risk or with established coronary heart disease [28], which is in line with our results. One of the possible explanations as to why no effects of PCSK9 inhibitors on inflammatory parameters were found in both studies is the background therapy with statins, because the majority of patients received maximally tolerated statin therapy. Statins have been shown to affect hsCRP, TNF-α, and IL6 levels in patients at high risk for CVD [29]. Indeed, statins, in addition to LDL-C, also lower inflammation, so PCSK9 inhibitors may not further reduce inflammatory markers [30,31]. Statins have a direct effect on various cell types and signaling pathways involved in the atherogenic process via lipid-lowering and also lipid-independent mechanisms, which contribute to the atheroprotective effects [32]. Baseline hsCRP was less than 1 mmol/L in our patients, which is considered low risk [33]. Despite the dramatic reduction in LDL-C levels, hsCRP remains a risk marker regardless of the LDL-C level achieved [34]. The same is true for IL6, as reported in the CANTOS trial. Namely, antagonism of IL1-reduced circulating IL6 levels [35]; however, the residual risk of CVD was proportional to IL6 levels in a subgroup of the CANTOS trial [36]. Interestingly, although the change in inflammatory markers after treatment with PCSK9 inhibitors was not significant, we found significant positive correlations between the change in TNF-α and IL6 levels, TNF-α and IL8 levels, and IL6 and IL8 levels after treatment with PCSK9 inhibitors. Therefore, there seems to be a mechanism that regulates cytokine levels, which may contribute to residual inflammation.

Similarly, in patients with elevated Lp(a) and LDL-C levels, treatment with the PCSK9 inhibitor evolocumab did not alter either local inflammation in the arterial wall or systemic inflammation measured by hsCRP, although LDL-C levels were reduced by more than 60% and Lp(a) by almost 14% [37]. Local inflammation was measured using 18F-fluoro-deoxyglucose positron-emission tomography/computed tomography (18F-FDG PET/CT). Arterial 18F-FDG uptake correlates with arterial macrophage content [37]. In this study, more than 50% of the patients were treated with statins. On the other hand, in patients with coronary artery disease or familial hypercholesterolemia who do not take statins due to statin intolerance, treatment with alirocumab attenuates arterial wall inflammation without changing hsCRP [38]. The difference between these two studies was higher Lp(a) levels both at baseline and the end of the study. Although we cannot directly compare these two studies, we can assume that this difference may explain the persistent arterial wall inflammation.

Patients after myocardial infarction have a higher risk of recurrent events, which has been associated with increased levels of proinflammatory molecules [39]. We showed that genetic variability in *CRP*, *TNF-α*, and *IL6* genes was not associated with the corresponding plasma levels at enrolment. Some previous studies also reported associations between CRP levels and polymorphisms in genes encoding *IL6* [40] and *TNFA* [41]. However, in our study, *TNFA* rs1800629 and *IL6* rs1800795 were not associated with significant differences in hsCRP levels, which is consistent with the AIRGENE study that included patients between 3 months and 6 years after myocardial infarction [42].

Besides genetic predisposition, chronic inflammation has also been shown to increase Lp(a) levels. Studies examining the effects of the *IL6* polymorphism in patients with rheumatoid arthritis, which has been linked to elevated Lp(a) levels, have shown that blocking the IL6 receptor, but not TNF-α signaling, decreased Lp(a) levels by 30–40% [43,44]. In addition, in patients with elevated Lp(a), the polymorphism *IL6* rs1800795 was associated with a significantly increased odds ratio of having substantially elevated serum Lp(a) levels [45]. The results were confirmed in patients without chronic inflammatory disease, suggesting a positive association between IL6 and Lp(a) levels [46]. Our results showed no significant differences in Lp(a) levels between the different here assessed genotypes, which may be due to the fact that only patients with very high Lp(a) levels were included. Moreover, treatment with the IL6 receptor antagonist had no effect on Lp(a) levels in patients with non-ST myocardial infarction, which was explained by background therapy with statins, because statins may increase Lp(a) levels [47]. In our study, all patients received maximally tolerated statin therapy, which may explain the lack of influence of variability in the *IL6* gene on Lp(a) levels.

In this context, a *post hoc* analysis of the SPIRE trial showed that the residual inflammatory risk persists after treatment with statins and PCSK9 inhibitor and contributes significantly to recurrent events [48], but the underlying mechanisms are not fully elucidated. We speculated that the modulation of inflammation by gene polymorphisms may play an important role.

The following limitations of our study should be considered. The main limitation is the small sample size, which is mainly due to the strict inclusion criteria because only patients with very high Lp(a) levels and premature myocardial infarction were included in the study. In addition, we studied only three SNPs. On the other hand, we consider the longitudinal design as one of the major strengths of our study, which allowed a chronological assessment of inflammatory status based on different genotypes. Moreover, the study was randomized and placebo controlled. Further studies are needed to identify genetic risk markers that could be included in the inflammatory gene score to predict inflammatory status for each individual [49].

## 5. Conclusions

The present study in patients with premature myocardial infarction and very high Lp(a) showed no association between the *CRP* rs1800947, *TNFA* rs1800629, and *IL6* rs1800795 polymorphisms and plasma levels of hsCRP, TNF-α, and IL6 at enrollment. Plasma levels of inflammatory markers also did not change after treatment with PCSK9 inhibitors. However, after 6 months of treatment with PCSK9 inhibitors, the *IL6*-174CC genotype was associated with a significant difference in IL6 levels, indicating the possible role in the regulation of inflammation. Further studies are needed to elucidate complex mechanisms underlying inflammatory imbalance in patients with established CVD and high risk for recurrent events, which would hopefully lead to better preventive and therapeutic strategies.

## Figures and Tables

**Table 1:** Baseline characteristics of patients included in the study (*n* = 69).

Parameter	Value
Age at inclusion (years)	52.6 (45.8–56.9)
Body mass index (kg/m^2^)	28.6 ± 3.9
Systolic blood pressure (mmHg)	128 (120–135)
Diastolic blood pressure (mmHg)	78 (70–82)
Heart rate (beat/min)	61 (56–67)
Total cholesterol (mmol/L)	4.24 ± 0.82
HDL-C (mmol/L)	1.20 ± 0.28
Non-HDL-C (mmol/L)	2.90 (2.40–3.68)
LDL-C (mmol/L)	2.27 (1.69–2.66)
Triglycerides (mmol/L)	1.43 (1.01–2.12)
Lp(a) (mg/L)	1483 (1196–1785)
ApoA1 (g/L)	1.28 (1.19–1.45)
ApoB (g/L)	0.79 (0.67–0.99)
hsCRP (mg/L)	0.87 (0.41–2.31)
TNF-α (ng/L)	3.91 (3.13–5.14)
IL6 (ng/L)	1.69 (1.20–2.18)
IL8 (ng/L)	13.5 (10.6–17.8)

HDL-C, high-density lipoprotein cholesterol; LDL-C, low-density lipoprotein cholesterol; Lp(a), lipoprotein (a); apo, apolipoprotein; hsCRP, high-sensitivity C-reactive protein; TNF-α, tumor necrosis factor-α; IL, interleukin. Data are presented as medians (25–75%) for variables not normally distributed and as means ± standard deviation for variables with normal distribution.

**Table 2:** Genotype and minor allele frequency of included patients for selected polymorphisms (*n* = 69).

SNP	Genotype	Number (%)	*p* Value HWE	MAF
*CRP* rs1800947	CC	58 (84.1)	1.000	0.087
	CG	10 (14.5)		
	GG	1 (1.4)		
*TNFA* rs1800629	GG	58 (84.1)	1.000	0.087
	GA	10 (14.5)		
	AA	1 (1.4)		
*IL6* rs1800795	CC	16 (23.2)	0.988	0.478
	GC	34 (49.3)		
	GG	19 (27.5)		

SNP, single nucleotide polymorphism; HWE, Hardy–Weinberg equilibrium; MAF, minor allele frequency.

**Table 3:** Plasma levels of inflammatory markers in control (*n* = 30) and treatment (*n* = 68) group at baseline and after 6 months.

Inflammatory Marker	Group	Baseline	After 6 Months	*p* Value
hsCRP (mg/L)	Control	0.80 (0.39–2.23)	0.74 (0.35–1.47)	0.713
	Treatment	0.85 (0.36–1.81)	0.79 (0.31–1.57)	0.989
TNF-α (ng/L)	Control	3.74 (2.99–5.02)	3.91 (3.05–5.25)	0.396
	Treatment	3.91 (3.36–5.36)	3.30 (2.53–6.04)	0.402
IL6 (ng/L)	Control	1.69 (1.19–2.18)	1.69 (1.19–2.75)	0.614
	Treatment	1.69 (1.20–2.53)	1.36 (1.16–2.18)	0.066
IL8 (ng/L)	Control	11.8 (10.8–16.1)	11.5 (10.8–16.3)	0.819
	Treatment	13.5 (10.7–17.7)	15.5 (10.8–24.1)	0.250

hsCRP, high-sensitivity C-reactive protein; TNF-α, tumor necrosis factor-α; IL, interleukin. Data are presented as median and interquartile range (25–75%).

**Table 4:** Association of SNPs with plasma levels of inflammatory markers at enrolment (*n* = 68).

SNP	Genotype	Median (25–75%)	*p* Value
*CRP* rs1800947	CC	0.93 (0.41–2.10) mg/L	0.664
	CG+GG	0.81 (0.40–2.38) mg/L	
*TNFA* rs1800629	GG	3.91 (3.05–5.14) ng/L	0.555
	GA+AA	3.83 (3.28–35.27) ng/L	
*IL6* rs1800795	CC	1.69 (1.20–2.07) ng/L	0.888
	CG+GG	1.69 (1.18–2.36) ng/L	

SNP, single nucleotide polymorphism.

**Table 5:** The association of genetic variability in inflammatory genes with the median level of Lp(a) (mg/L) at enrolment (*n* = 69).

SNP	Genotype	Median (25–75%)	*p* Value
*CRP* rs1800947	GG+GC	1559 (974–1782)	0.782
	CC	1463 (1196–1837)	
*TNFA* rs1800629	AA+AG	1607 (1219–2105)	0.376
	GG	1462 (1171–1741)	
*IL6* rs1800795	GG+CG	1461 (1011–1785)	0.316
	CC	1580 (1295–1871)	

SNP, single nucleotide polymorphism.

**Table 6:** Spearman’s Rho correlation analysis between changes in inflammatory marker and lipid levels after treatment with PCSK9 inhibitors.

Parameter	hsCRP		TNF-α		IL6		IL8	
	Rho	*p* Value	Rho	*p* Value	Rho	*p* Value	Rho	*p* Value
Total cholesterol	−0.022	0.863	−0.081	0.519	0.061	0.629	0.056	0.656
HDL-C	−0.138	0.272	−0.037	0.769	0.091	0.471	−0.015	0.904
LDL-C	0.044	0.726	−0.009	0.946	0.103	0.415	0.087	0.489
Triglycerides	−0.019	0.881	−0.097	0.443	−0.077	0.543	−0.077	0.543
Lp(a)	0.104	0.419	−0.168	0.188	−0.227	0.073	−0.076	0.552
ApoB	0.009	0.945	−0.097	0.453	0.031	0.808	0.064	0.623
ApoA1	−0.116	0.371	−0.166	0.198	−0.077	0.549	0.098	0.449

hsCRP, high-sensitivity C-reactive protein; TNF-α, tumor necrosis factor-α; IL, interleukin; HDL-C, high-density lipoprotein cholesterol; LDL-C, low-density lipoprotein cholesterol; Lp(a), lipoprotein(a); apo, apolipoprotein.

## Data Availability

The data presented in this study are available upon request from the corresponding author.

## Abbreviations

ApoA1	apolipoprotein A1
ApoB	apolipoprotein B
CVD	cardiovascular disease
HDL-C	high-density lipoprotein cholesterol
HWE	Hardy-Weinberg equilibrium
hsCRP	high-sensitivity C-reactive protein
IL	interleukin
LDL-C	low-density lipoprotein cholestrerol
Lp(a)	lipoprotein(a)
MAF	minor allele frequency
PCSK9	proprotein convertase subtilisin/kexin type 9
SNP	single nucleotide polymorphism
TNF-α	tumor necrosis factor-α
